# Extract of *Acalypha australis L.* inhibits lipid accumulation and ameliorates HFD-induced obesity in mice through regulating adipose differentiation by decreasing PPARγ and CEBP/α expression

**DOI:** 10.29219/fnr.v65.424

**Published:** 2021-03-01

**Authors:** Lang You, Fengxia Li, Yan Sun, Liang Luo, Jian Qin, Tao Wang, Yuchen Liu, Ruogu Lai, Ruohan Li, Xiaoran Guo, Qiuyan Mai, Yihang Pan, Jianrong Xu, Ningning Li

**Affiliations:** 1 School of Life Science and Engineering, Southwest University of Science and Technology, Mianyang, Sichuan, China; 2 Tomas Lindahl Nobel Laureate Laboratory, Precision Medicine Research Centre, The Seventh Affiliated Hospital of Sun Yat-sen University, Shenzhen, Guangdong, China; 3 Department of Traditional Chinese Medicine, The Seventh Affiliated Hospital of Sun Yat-sen University, Shenzhen, Guangdong, China; 4 Department of Critical Care Medicine, The Seventh Affiliated Hospital of Sun Yat-sen University, Shenzhen, Guangdong, China; 5 Department of Rheumatology and Immunology, Guangdong Provincial People’s Hospital, Guangdong Academy of Medical Sciences, Guangzhou, China

**Keywords:** potherb, Acalypha australis L., obesity, 3T3-L1, adipogenesis

## Abstract

**Background:**

Obesity is a principal risk factor for the development of type 2 diabetes and cardiovascular diseases. Natural plants and/or foods play an important role in the management of obesity. *Acalypha australis L.* (AAL) is a kind of potherb popular among Asian populations, and it is also consumed as a food ingredient and traditional herbal medicine.

**Objective:**

We investigated the effects of water extract from AAL on high-fat-diet (HFD)-induced obese mice and 3T3-L1 adipocytes to develop a new functional food material.

**Design:**

Nine-week-old male mice were randomly divided into control (chow diet, *n* = 6) and HFD (*n* = 30) group. From 12-weeks onward, mice in the HFD group were further separated into model (saline, 6 mL/kg), simvastatin (0.11 mg/mL, 6 mL/kg), and AAL treatment (low, middle, and high dosage: 300, 600, and 900 mg/kg) group, with 6 animals per group, while mice in the control group were treated with saline (6 mL/kg). Food intake, body/fat weight, liver/kidney indexes, and lipid profiles were determined. Tissues were fixed with formalin for pathological examination. Western blotting and PCR were performed to evaluate the protein and mRNA expression in 3T3-L1 adipocytes. Oil Red O staining was used to determine lipid accumulation.

**Results:**

AAL administration significantly suppressed body weight gain, and reduced fat pad weight and Lee’s index in obese mice, but had no effect on liver/kidney index. AAL also reduced serum cholesterol, triglyceride, and LDL-C and increased HDL-C levels. Histological analysis revealed that AAL significantly ameliorated lipid accumulation in the liver and subcutaneous adipose tissue. *In vitro*, Oil Red O staining showed that AAL inhibited adipose differentiation by down-regulating the gene and protein expression of PPARγ and C/EBPα. AAL also reversed HFD-induced intestinal dysbacteriosis.

**Conclusion:**

AAL water-soluble extract has a significant anti-adipogenic effect in the HFD-induced obese mice model.

## Popular scientific summary

- This is the first report detailing the potential anti-obesity effect of *Acalypha australis L.* (AAL).
- The beneficial effects of AAL were mediated through regulating adipogenesis by suppressing PPARγ and CEBP/α expression during adipose differentiation.
- AAL also improved obesity-related hyperlipidemia, possibly through remodeling high-fat-diet (HFD)-induced enteric dysbacteriosis.

Obesity is defined as excessive fat accumulation that might impair health and is diagnosed at a BMI ≥ 30 kg/m^2^ (1). According to WHO, the worldwide prevalence of obesity nearly tripled between 1975 and 2016. In 2016, 13% of the world’s adult population (11% of men and 15% of women) were classified as obese (2). As a worldwide heath problem, obesity substantially increases the risk of diseases such as type 2 diabetes mellitus, fatty liver disease, and hypertension, thereby contributing to a decline in the quality of life as well as life expectancy (1). Treatment options for obesity are abundant including lifestyle intervention, drugs, surgery, and behavioral therapies (3); of the aforementioned interventions, administration of anti-obesity drugs features with high compliance. However, unexpected side effects often manifest. Some of the anti-obesity drugs have even been withdrawn from the market, such as Redux (4) and Pondimin (5). Given the increasing demand for anti-obesity measures, foods and natural plants emerge as important players in the management of obesity.

*Acalypha australis L.* (AAL), also known as copperleaf, grows in humid plains in most parts of Asia and is one of the potherbs popular among Asian populations. In China, AAL is also used as a traditional herbal medicine for treating diseases like diarrhea and snakebite. It has been reported that AAL has multiple pharmacological functions such as anti-inflammatory (6), anti-apoptosis (7), detoxification, and cough relieving (8). Experimental studies demonstrated that AAL improved trinitro-benzene-sulfonic acid (TNBX)-induced ulcerative colitis in rats by inhibiting inflammatory cell infiltrates, and decreasing the MPO activity and frequency of diarrhea (9). Three active chemical compounds that have been identified from AAL show efficacy of ameliorating ulcerative colitis, including 4-hydroxybenzoic acid methyl ester, 3-methoxy-4-hydroxybenzoic acid, and protocatechuic acid (10). AAL, together with berberine, is the main ingredient of Xiancai Huangliansu Capsule, which is used to treat diarrhea (11). It has been reported that extracts from AAL are rich in flavonoids (12). Studies have demonstrated that flavonoids were effective in blood lipid adjustment and obesity reversion (13, 14). However, little direct research concerning the effects of AAL on obesity or lipid metabolism has been performed.

Obesity arises when an individual’s energy intake rate exceeds energy consumption rate. At the cellular level, obesity is the result of an increase in the number or volume of individual fat cells. Proliferation, differentiation, and apoptosis of preadipocytes affect the number of adipocytes, and the volume of adipocytes mainly depends on the balance between lipid formation and lipid hydrolysis (15). The differentiation of preadipocytes into mature adipocytes is regulated by a series of transcription factors such as PPARγ and C/EBPα (16, 17). Gut microbiota also play a critical role in human energy metabolism. Microbes can harvest energy from indigestible dietary substances, while their metabolites have different effects on the host (18). As a potential target for reducing obesity, the intestinal flora can be regulated by natural products and/or food constituents (19).

In this study, we evaluated the anti-obesity properties and the underlying mechanism of AAL extract in the high-fat-diet (HFD)-induced obese mouse model and in 3T3-L1 cells, thus exploring the possibility of developing AAL into a new functional food supplement.

## Materials and methods

### AAL extract preparation

We identified and collected AAL according to the previous literatures (20, 21). After collection, the samples were washed with distilled water, and the moisture was removed at 60°C. Dried plants were pulverized and sifted through a 0.5 mm sieve until powder was obtained. The sample was extracted with 8 times (w/v) distilled water for 2 h, and the filtrate was collected. After repeating the extraction step twice, the three filtrates were combined and then concentrated on a rotary evaporator at 80°C to a 1 g/mL paste and stored at 4°C.

### Total flavonoid determination by spectrophotometry

Standards (Rutin, SR8250, Solarbio) and samples were dissolved in 60% ethanol. Thereafter, 5% sodium nitrite and 10% aluminum nitrate solution were added to the standard and sample wells, and the solution was incubated at room temperature for 15 min. The absorbance was measured at 510 nm, and the total flavonoid content was calculated according to the standard curve.

### Animals

Nine-week-old KM male mice (SPF grade, 20 ± 2 g) were purchased from Dashuo Laboratory Animal Co., Ltd. (Sichuan, China). All procedures involving animals were approved by the Animal Ethics Committee of the School of Life Science and Engineering, Southwest University of Science and Technology (20180001). Before the experiment, the mice were adapted to the conditions for 1 week. Animals were housed in standard laboratory conditions: 12: 12 h light: dark cycle, 24 ± 1°C temperature with *ad libitum* food and water unless otherwise stated.

### Experimental design

**Fig. 1:**
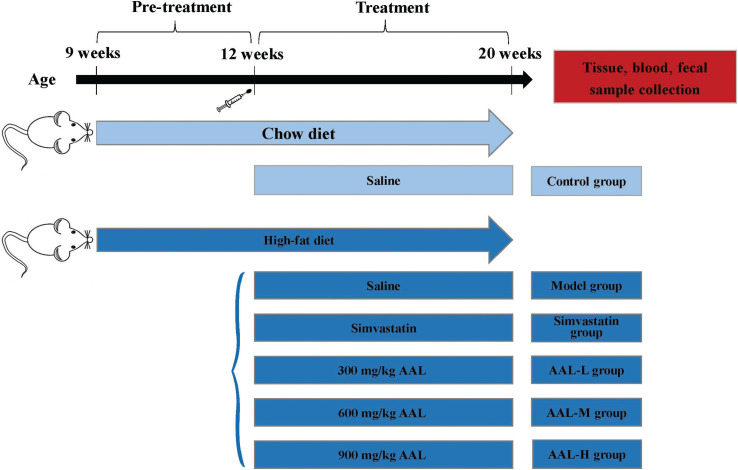
Experimental time lines.

Thirty-six KM male mice were randomly divided into HFD group (*n* = 30) and standard chow diet group (STD, *n* = 6). Body weight was measured every 3 days. From 9-weeks onward, mice in the STD group were fed with chow diet, while the ones in the HFD groups were fed with HFD for 3 weeks. HFD-induced obese mice model was established when the average weight of mice in the HFD group increased more than 20% of the ones in the STD groups (22). Thereafter, HFD-fed mice were randomly distributed into five groups: (1) 300 mg/kg AAL group (low-dose treatment, AAL-L); (2) 600 mg/kg AAL group (medium-dose treatment, AAL-M); (3) 900 mg/kg AAL group (high-dose treatment, AAL-H); (4) simvastatin positive control group (simvastatin); and (5) high-fat group with saline treatment (Model). Body weight and food intake were recorded daily for 8 weeks. Body weight was measured at fixed times every 3 days. Food intake was weighed daily by subtracting the weight of food pellets remaining on the day of the measurement from the amount of the previous day. Prior to sacrifice, mice were fasted for 12 h, the body weight and length of the mice were measured, and the Lee’s index was calculated. The schedule and processing of the animal experiments is shown in Fig. 1.

The normal diet (chow diet) was purchased from Dashuo Laboratory Animal Co., Ltd. (Sichuan, China), while the HFD was prepared by the laboratory according to the feed formula and methods of the local provincial animal experimental center. The composition of HFD is shown in Supplementary Table 1.

### Biochemical parameter analysis

At the end of the experiment, blood was taken retro-orbitally after the mice received 1% sodium pentobarbital (0.5 mL/100 g) anesthesia. Blood samples were then centrifuged at 2,500 rpm for 10 min at 4°C, and serum was collected and stored at –80°C. Mice were sacrificed by cervical dislocation. Serum total cholesterol (TC), triglyceride (TG), low-density lipoprotein-cholesterol (LDL-C), and high-density lipoprotein-cholesterol (HDL-C) were measured using the assay kit (Jiancheng Bioengineering Institute, Nanjing); alanine aminotransferase (ALT) and aspartate aminotransferase (AST) levels were determined using the assay kit (Jiancheng Bioengineering Institute, Nanjing).

### Histological analysis

Liver, kidney, perirenal, and epididymal adipose tissue samples were collected, washed with saline, weighed, and stored at −80°C. Additional liver tissue was collected, fixed in 4% formalin for 24 h, and then embedded in paraffin. The thickness of paraffin tissue slices is 5 μm, which were then stained with hematoxylin–eosin. Paraffin sections are dewaxed in xylene for 5–10 min and then in fresh xylene for another 5–10 min. After that, they were in absolute ethanol for 5 min, 90% ethanol for 2 min, 80% ethanol for 2 min, 70% ethanol for 2 min, and distilled water for 2 min. Then they were stained with hematoxylin staining solution for 5–10 min, washed with tap water for 10 min and distilled water for 10 sec before differentiating with 1% hydrochloric acid alcohol for 20 s, and washed with tap water for 30 s. Next, they were stained with eosin staining solution for 2 min and washed with tap water for 30 s. They were placed in 70% ethanol for 10 s, 80% ethanol for 10 s, 90% ethanol for 2 min, and absolute ethanol for 2 min. Finally, paraffin sections were in xylene for 5 min and then in fresh xylene for 5 min, and sealed by a neutral gum. Images of the tissue sections were taken using a DM48 microscope (Leica, Germany) at 200× magnifications.

After staining adipose tissues with hematoxylin and eosin, images were taken with DM48 microscope (Leica, Germany) at 200× magnifications. Three out of 18 images were randomly selected for analyses, and the number and diameter of adipocytes in each field were measured and averaged.

### 3T3-L1 cells experiment

#### Cell culture

3T3-L1 cell lines were obtained from the Shanghai Institute of Chinese Academy of Sciences (CAS). Cells were grown in high-glucose DMEM (Gibco, United States) supplemented with 10% fetal bovine serum (FBS, Australia) and 1% antibiotic antimycotic solution (Solarbio, United States) before differentiation. Cells were maintained at 37°C in a humidified incubator with 5% CO_2_, with culture medium being changed every 2 days.

#### Cell viability assay

5 × 10^3^ cells per well in 100 μL medium were seeded into 96-well plates and incubated at 37°C in a humidified incubator with 5% CO_2_ for 24 h. DMEM cell basal medium was replaced with DMEM mixed with AAL extracts of different concentrations, and a blank group was established and incubated for 24 h; DMEM cell basal medium was set as blank group and incubated for 24 h; 10 μL CCK8 (Beyotime, China) was added into the wells, followed by 4 h incubation at 37°C. Absorbance was measured by microplate reader (BIO-RAD, United States) at 450 nm. Growth curves were plotted according to the OD value of each well.

#### Differentiation

Cells were harvested by trypsinization and then seeded into the desired culture vessel and left to reach 100% confluence. After 48 h, the growth medium was replaced with a differentiation medium (10% FBS), 3-isobutyl-1-methylxanthine (0.5 mmol/L), dexamethasone (1 μmol/L), and insulin (10 μg/mL) for incubation for another 48 h. Then the differentiation medium was replaced with an adipocyte maintenance medium containing 10% FBS and insulin (10 μg/mL). The adipocyte maintenance medium was changed every 48 to 72 h. The cells were fully differentiated between 7 to 15 days after induction as evidenced by lipid droplet formation.

#### Oil Red O staining

The cell culture medium was removed from the differentiated 3T3-L1 cells and washed twice with PBS. Follow the instructions for Oil Red O (ORO) staining solution (Cell-Specific) Kit (Solarbio, China). Add ORO fixative fixation solution to the cells for 20–30 min and then discard the fixation solution and wash it twice with distilled water; 60% isopropanol was added for 5 min and then discarded. Add freshly prepared ORO stain, stain for 10–20 min, discard the staining solution, and wash with distilled water 2–5 times until there is no excess staining solution. Add Mayer hematoxylin staining solution, stain for 1–2 min, then discard the staining solution, and wash with distilled water 2–5 times. Add ORO buffer for 1 min and discard. Add distilled water to cover the cells. Images were acquired with CKX53 light microscope (Olympus, Japan) and analyzed using Image J (Image J V1.8.0.112, United States). Three images were randomly selected to evaluate the percentage of lipid area.

#### BODIPY staining of lipid droplets

After 7 days of differentiation, 3T3-L1 cells treated with saline, simvastatin, and AAL of different dosages were stained with BODIPY and DAPI. After the cell culture medium was removed, differentiated 3T3-L1 cells were rinsed 3 times with 1× PBS (TRAN, China), fixed with 4% formaldehyde for 15 min, and rinsed with 1× PBS (TRAN, China) again. Cells were sequentially stained with BODIPY 493/503 (Invitrogen, stock concentration 5 mM, working solution 1: 2,500 dilution) for 15 min at room temperature and then washed 3 times with 1× PBS (TRAN). Lipid accumulation was observed using DMI8 fluorescent inverted microscope (Leica, Germany) in cells with DAPI (BioLegend, stock concentration 10 mM, working solution 1: 10,000 dilution) stained nuclei.

### Western blot

After the cells were cultured to 70–80% confluence, the medium was removed, and the cells were washed 3 times with PBS. RIPA (Beyotime, China) and protease inhibitor (Thermo Fisher Scientific, United States) were added to the cells and left at 4°C for 30 min. Total protein was centrifuged at 12,000 rpm for 15 min and stored at –80°C. The BCA method was used to determine the protein concentration. Cellular proteins were electroblotted onto a polyvinylidene fluoride membrane following separation via SDS-PAGE. Blots were incubated for 1 h with blocking solution (5% skim milk in TBS, T/TBS) at room temperature, followed by overnight incubation with anti-PPAR gamma (ab209350, Abcam) or anti-CEBP Alpha (ab15047, Abcam) at 4 °C. Blots were washed three times with Tween 20/Tris-buffered saline (T/TBS) and incubated with horseradish peroxidase-conjugated secondary antibody (1: 3,000, Asbio, China) for 2 h at room temperature. Blots were washed three times with T/TBS and then developed via enhanced chemiluminescence (Bio-Rad, USA). Data were done in triplicate. Gray value was calculated by using Quantity One Software (version 4.6.6).

### RT-PCR

mRNA expression level of PPARγ, C/EBPα, and aP2 was measured. The total cellular RNA was isolated by Trizol Reagent (Thermo Fisher, USA) after cells were treated with different concentration of AAL for 24 h. RNA was reverse transcribed using an Evo M-MLV RT Premix for qPCR (AG, China). Real-time reverse transcription PCR was performed using SYBR® Green Premix Pro Taq HS qPCR Kit (AG, China). GAPDH was used as internal housekeeping control gene for normalization and quantification of PPARγ, C/EBPα, and aP2 expression. Primer sequences are listed in Supplementary Table 2. Relative quantification of mRNA expression was evaluated using the comparative cycle threshold (Ct) method. All experiments were done in triplicate. Mean normalized gene expression ± SEM was calculated from three independent experiments.

### Intestinal flora analysis

After the establishment of the obesity model, mice were treated with AAL extract, and the feces collection was performed every 4 weeks at the beginning of the first week; 1–2 g of feces were collected from each mouse for easy centrifugation in a tube and stored at –80°C. Take 1 g of feces from each group, add 4 mL of sterilized water, mix and dilute to different gradients (1 × 10^-5^, 1 × 10^-6^, 1 × 10^-7^). The bacterial solid was cultured, and after 24 to 72 h of culture, the microbial colony count was performed using a plate counting method. The results were counted in colony forming units (CFU/g), and the results were expressed as logarithmic values of the number of bacterial colonies per gram of feces.

### Statistical analysis

Differences between groups were analyzed by one-way analysis of variance (ANOVA). Data were shown as means ± SEM. Turkey’s post hoc test of GraphPad Prism version 7.0 software packages was used to perform statistical analyses. A value of *P* < 0.05 was considered as statistically significant.

## Results

### In vivo

#### Food intake, body weight changes, and fat ratio

To evaluate AAL effect on obesity, 9-week-old mice were fed with HFD. Food intakes and body weight changes recorded during AAL administration are shown in Table 1 and Supplementary Fig. 1. Compared to the control group, the body weight of mice in the model group increased significantly (*P* < 0.01), while the weights of the simvastatin-treated mice were significantly decreased, compared to the model group (*P* < 0.01). AAL-H showed similar effect as simvastatin, which remarkably reduced the body weight when compared to the model group (*P* < 0.01). In AAL-M and AAL-H group, comparing to the model group, there was a trend towards decrease in the body weight gain; however, it’s not significant. As for food intake, we could observe a significant increase in the model group (*P* < 0.01). Simvastatin significantly suppressed the appetite (*P* < 0.01), while there were no significant differences between the AAL groups.

**Table 1:** Dietary intake and animal weight during the treatment period

Groups	Dietary intake (g/day)	Weight during administration (g)
Control	6.61 ± 0.1	39.88 ± 0.8
Model	7.01 ± 0.2**	43.50 ± 1.6**
Simvastatin	6.53 ± 0.2^##^	39.75 ± 1.5^##^
AAL-L	7.02 ± 0.2	41.98 ± 0.9
AAL-M	7.15 ± 0.3	41.73 ± 1.2
AAL-H	6.91 ± 0.1	40.80 ± 0.7^##^

Notes: **P < 0.01, Model vs Control;

## *P* < 0.01, model vs. simvastatin, AAL-L, AAL-M, AAL-H. Data are presented as means ± SEM, *n* = 6.

**Fig. 2:**
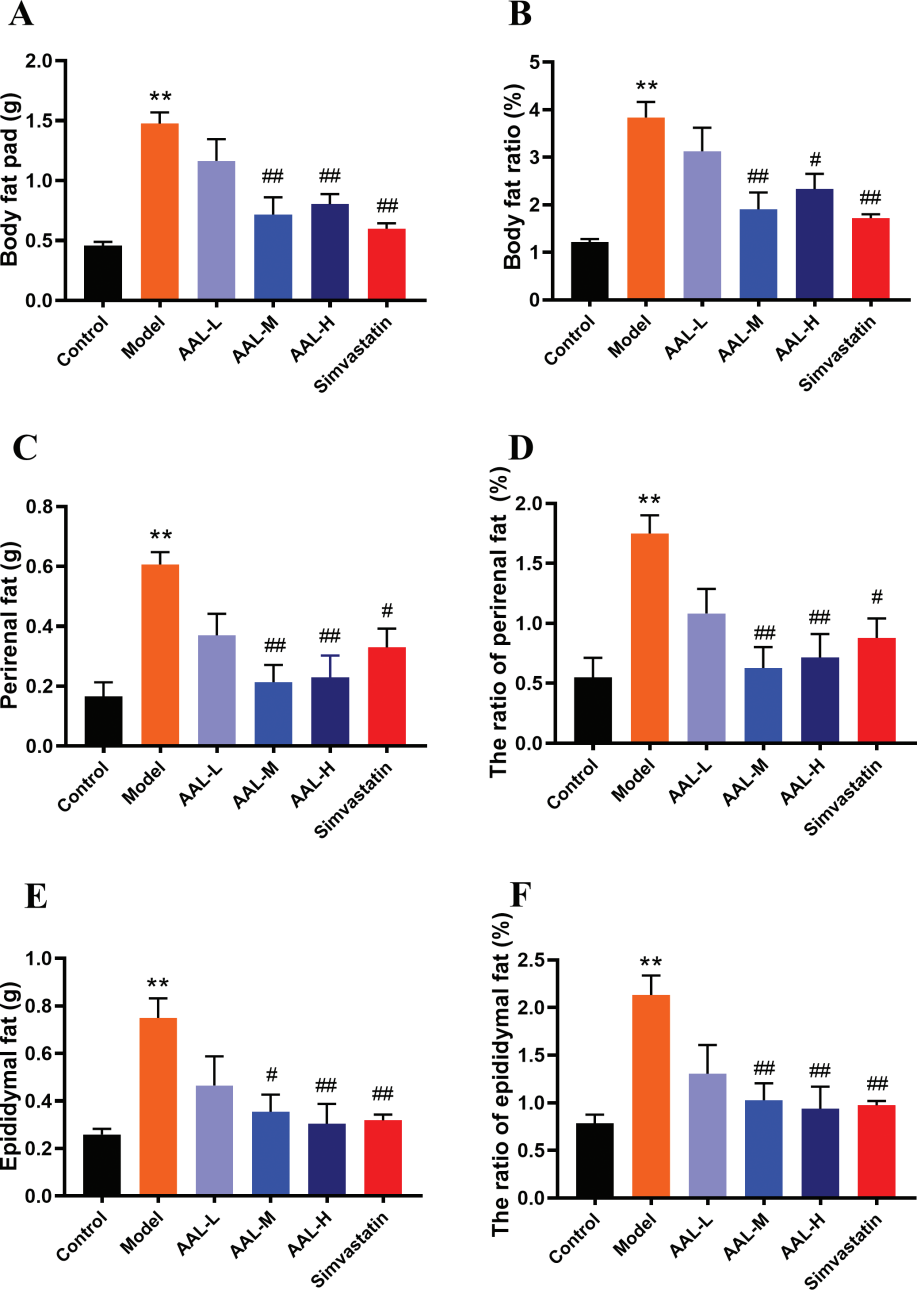
AAL reduces adipose tissue weight. Body fat pad (A), perirenal fat (C), epididymal fat (E), the ratio of body fat (B), perirenal fat (D) and epididymal fat (F). **P < 0.01 compared with control group. ^#^*P* < 0.05*,* ^##^*P* < 0.01 compared with model group. Data are presented as means ± SEM, *n* = 6.

To further investigate the effect of AAL on obese mice, the body fat content was analyzed. In Fig. 2, the body fat ratio in the model group was significantly higher than that in the control group (*P* < 0.01). Simvastatin significantly inhibited fat accumulation, and AAL showed similar effect as simvastatin (*P* < 0.01). Perirenal and epididymal fat of the model group increased significantly (*P <* 0.01). The weight of the perirenal and epididymal fat was significantly reduced after AAL or simvastatin treatment (*P* < 0.01; Fig. 2C, D, E and F). These results demonstrate that AAL extract can inhibit fat accumulation in HFD-fed mice.

#### AAL significantly reduces Lee’s Index but has no effect on Liver Index or Kidney Hypertrophy Index

Next, we determined the organ index of the mice at the end of the treatment. Liver Index, Kidney Hypertrophy Index, and the Lee’s Index can indicate the degree of obesity in mice (23). As shown in Table 2, there were no differences between groups in Liver Index and Kidney Hypertrophy Index. The Lee’s Index, on the other hand, was significantly elevated in the model group compared to all other groups (*P* < 0.05). Compared with the simvastatin group, only AAL-L group had lower Lee’s Index (*P* < 0.05), and there was no significant difference between simvastatin and AAL-M or AAL-H group.

**Table 2:** Effects of different concentrations of AAL on organ indexes

Groups	Lee’s index	Liver index	Kidney hypertrophy index (KI)
Control	205.31 ± 1.7	4.24 ± 0.3	1.57 ± 0.3
Model	220.81 ± 2.9*	4.82 ± 0.4	1.37 ± 0.2
Simvastatin	208.90 ± 2.4^#^	4.55 ± 0.7	1.53 ± 0.4
AAL-L	214.10 ± 1.9^#^^+^	4.49 ± 0.7	1.30 ± 0.1
AAL-M	209.30 ± 1.1^#^	4.70 ± 0.2	1.50 ± 0.3
AAL-H	204.87 ± 2.0^#^	4.65 ± 0.7	1.47 ± 0.2

Notes: *P < 0.05, control vs. model;

# *P* < 0.05, model vs. simvastatin, AAL-L, AAL-M, AAL-H;

+ *P* < 0.05, simvastatin vs. AAL-L, AAL-M, AAL-H. Data are presented as means ± SEM, *n* = 6.

#### AAL water extract’s effect on serum biochemical parameters

**Fig. 3:**
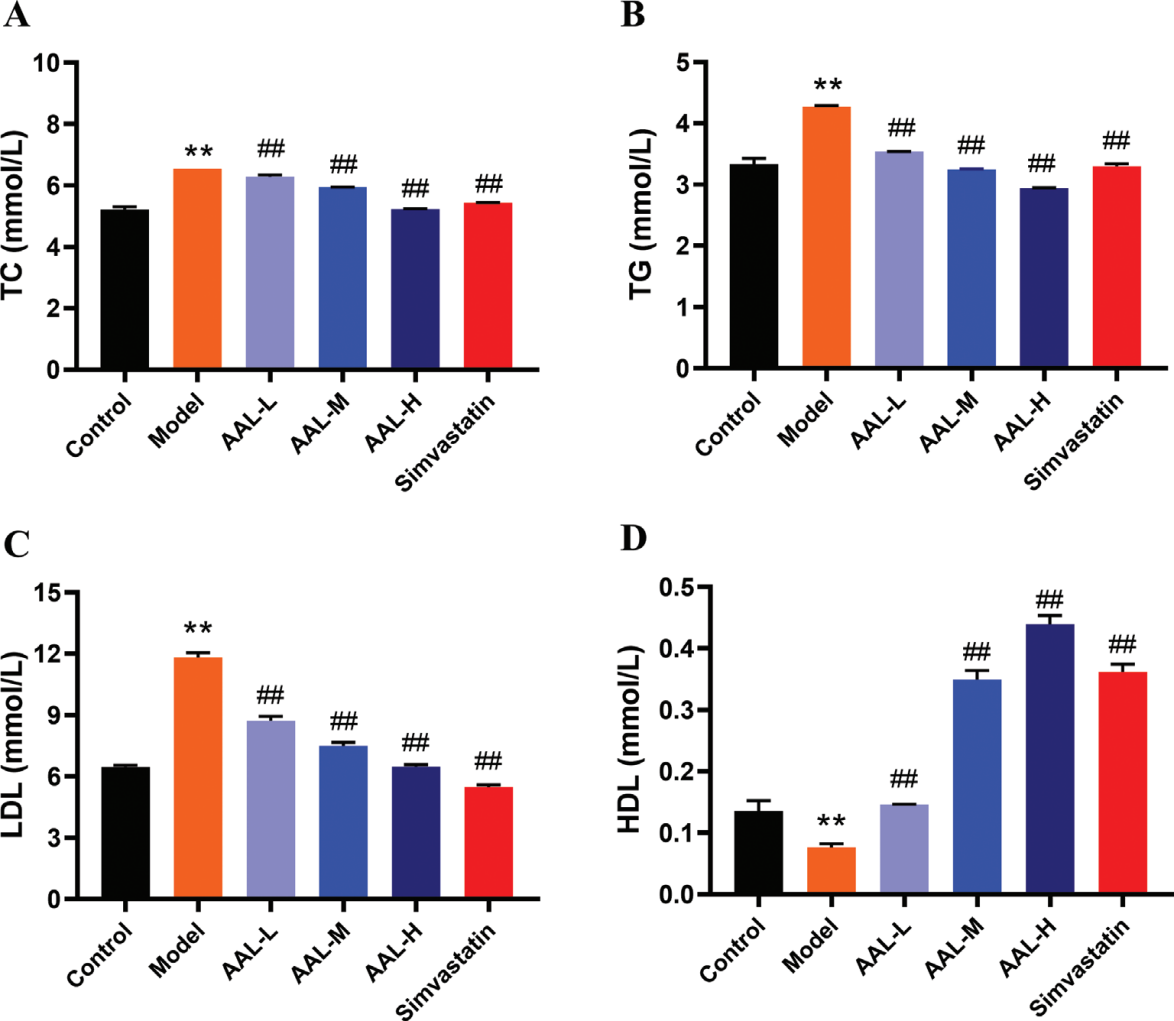
AAL ameliorates hyperlipidemia. The protein levels of the TC (A), TG (B), LDL (C) and HDL (D) in serum. **P < 0.01 compared with Control group. ^#^*P* < 0.05*,* ^##^*P* < 0.01 compared with Model group. Data are presented as means ± SEM, *n* = 6.

To investigate whether the treatment could regulate lipid composition, serum TG, TC, LDL, HDL, AST, and ALT levels were measured by assay kit. As shown in Fig. 3 and Supplementary Fig. 2, levels of TC, TG, and LDL were significantly increased in the model groups when compared to the control group (*P* < 0.01), implying that the experimental hyperlipidemic mouse model was successful. Compared to the model group, the serum levels for AAL-L, AAL-M, and AAL-H treatment groups were significantly decreased by 5, 10, and 20% for TC; by 18, 25, and 32% for TG; and by 17, 37, and 46% for LDL-C (*P* < 0.01), respectively. Moreover, the model group showed lower HDL levels than the control group (*P* < 0.01). The levels of HDL in the simvastatin, AAL-L, AAL-M, and AAL-H groups were higher than those in the model group (*P* < 0.01) and increased significantly by 374, 91, 356, and 476%, respectively. The results indicated that AAL water extract could reduce the serum lipid levels in obese mice.

#### Histopathological analysis of hepatic and adipose tissue

**Fig. 4:**
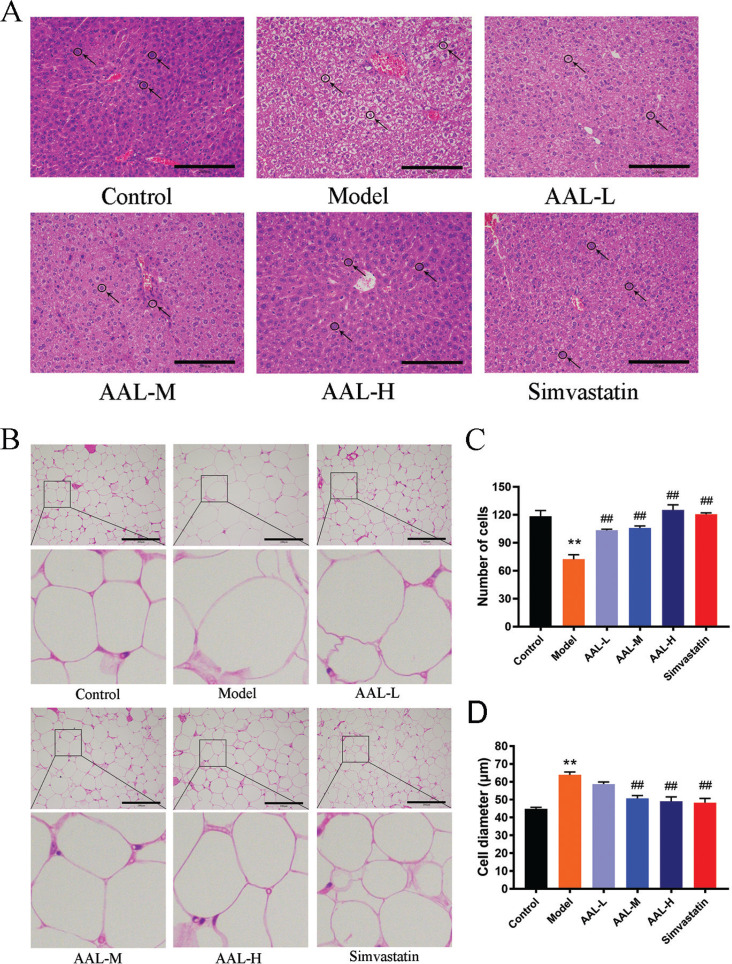
AAL treatment attenuates liver steatosis and affects adipose tissue morphology. Representative image of liver tissue (A). Representative image of the adipocyte tissues (B). All sections were stained with hematoxylin and eosin (H &E), red arrows indicate micro-vesicular fats. Number of adipocytes in the image (C). Adipocytes diameter in the image (D). Scale bar in all images is 100 μm. **P < 0.01 compared with control group. ^##^*P* < 0.01 compared with model group. Data are presented as means ± SEM, *n* = 6.

To investigate the effect of ALL treatment had on liver structure, histopathological staining of the liver was performed, and results are shown in Fig. 4A. The hepatocyte size was uniform, arranged neatly in the control group, and the liver tissues in the model group developed a high degree of steatosis, characterized by hepatocytes with severe fat vacuoles and the infiltration of inflammatory cells. Fat vacuoles around the hepatocyte nucleus were smaller in simvastatin, ALL-L, ALL-M and ALL-H groups when compared to the model group. Histopathology of perirenal and epididymal fat tissues is shown in Fig. 4B. Fat cells were located at the margin, and the cells are vacuolated. Average diameter of adipocytes was increased in the model group compared to the control group. The simvastatin, ALL-L, ALL-M, and ALL-H groups showed a significantly reduced average diameter of adipocytes compared with the model group, suggesting that the extract of AAL alleviated hepatic lipid accumulation and reduced the average diameter of adipocytes.

### In vitro

#### The effect of AAL on 3T3-L1 cells morphology and cell viability

**Fig. 5:**
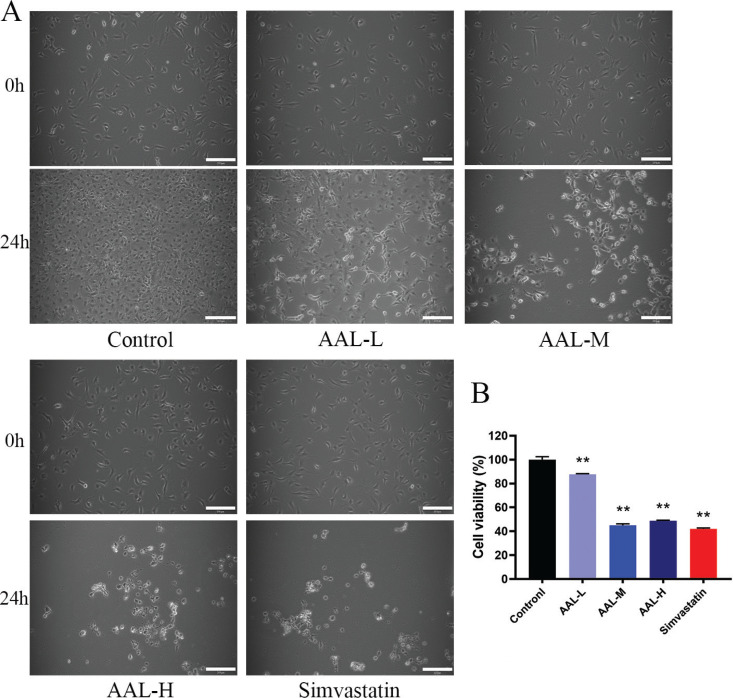
AAL affects 3T3-L1 cells morphology and cell viability. Effects of AAL on the morphology of 3T3-L1 cells after 0 and 24 h of treatment (A). Effect of AAL on 24 h viability of 3T3-L1 cells (B). **P < 0.01 compared with control group. Data are presented as means ± SEM.

First, we investigated whether AAL influences 3T3-L1 cell morphology. Cell morphology was analyzed following the incubation of 3T3-L1 cells with AAL extract at low (AAL-L, 0.5 mg/mL), medium (AAL-M, 1 mg/mL), and high (AAL-H, 2 mg/mL) concentration for 24 h. Simvastatin (10 μmol/L) was used as a positive control group. In Fig. 5, at 0 h, the 3T3-L1 cells had a clear boundary and the morphology showed a spindle-like shape. During the next 24 h, 3T3-L1 cells in the control group showed growth in a lot of cells. However, the simvastatin and AAL treatment groups showed that 3T3-L1 cells changed morphology from spindle-shaped to round-shaped, and the number of cells decreased.

The viability of 3T3-L1 cells was determined after 24 h. Compared with the control group, the viability of 3T3-L1 cells in the simvastatin group had 50% decrease, while the AAL-L group had 20% decrease. The cell viability of the AAL-M and AAL-H groups was similar to the simvastatin group, which had a 50% decrease.

#### AAL inhibits lipid accumulation

**Fig. 6:**
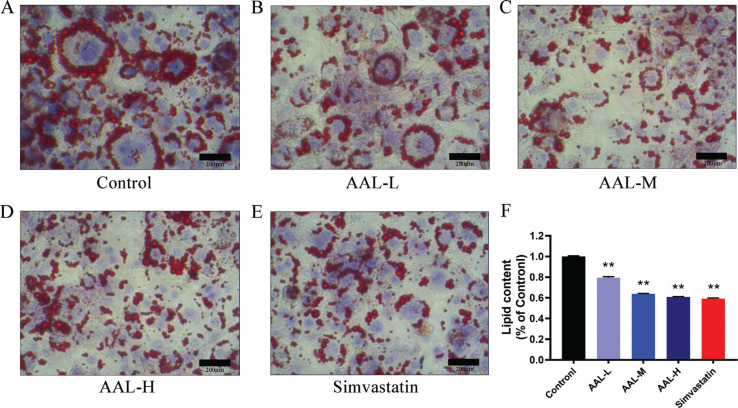
Lipid accumulations in 3T3-L1 adipocytes after AAL treatment. A, B, C, D, E for control, AAL-L, AAL-M, AAL-H, and simvastatin group, respectively. F showed the relative lipid content of each groups. **P < 0.01 compared with control group. Data are presented as means ± SEM.

**Fig. 7:**
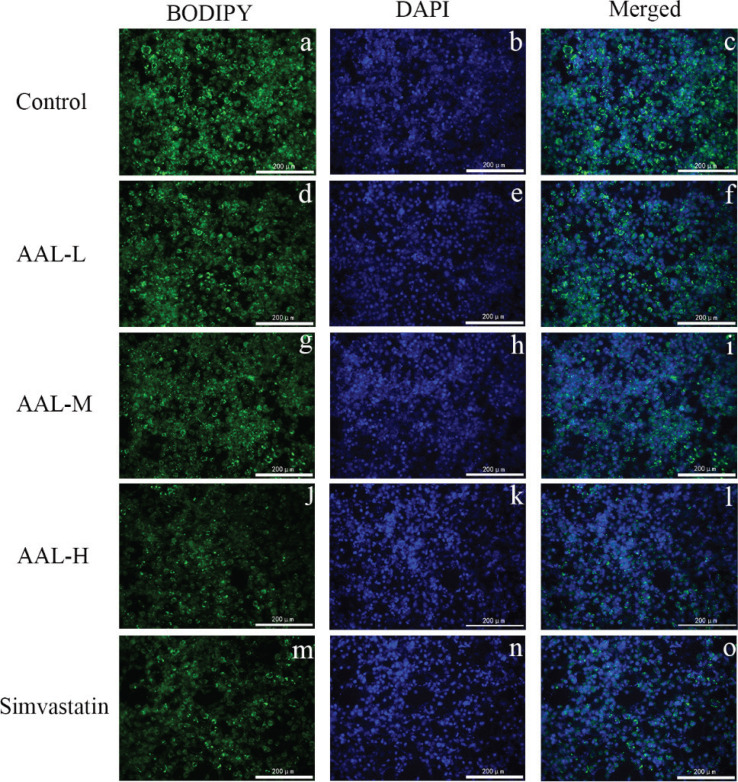
Fluorescence merged image of differentiated 3T3-L1 cells treated with saline, simvastatin, or AAL of different dosage. The representative fluorescence images were obtained using a 20× objective on DMI8 fluorescent inverted microscope. Lipid droplets and nuclei were visualized by using BODIPY 493/503 and DAPI, respectively, and merged together. Control, a–c; AAL-L, d–f; AAL-M, g–i; AAL-H, j–l; simvastatin, m–o. Scale bar, 200 μm.

Differentiated 3T3-L1 adipocytes are useful model for screening compounds that regulate lipid accumulation. In Fig. 6, the ORO experiment showed that cells in the control group had large lipid droplets and the highest lipid accumulation. Lipid accumulation was reduced by approximately 40% in simvastatin group. In addition, AAL treatment also showed an inhibitory effect on lipid droplet size, and the inhibitory effect of the AAL-M and AAL-H groups was like the simvastatin group (Fig. 6). BODIPY staining of the lipid droplets also showed a similar result (Fig. 7).

#### AAL suppressed the expression of C/EBPα and PPARγ in 3T3-L1 preadipocytes

**Fig. 8:**
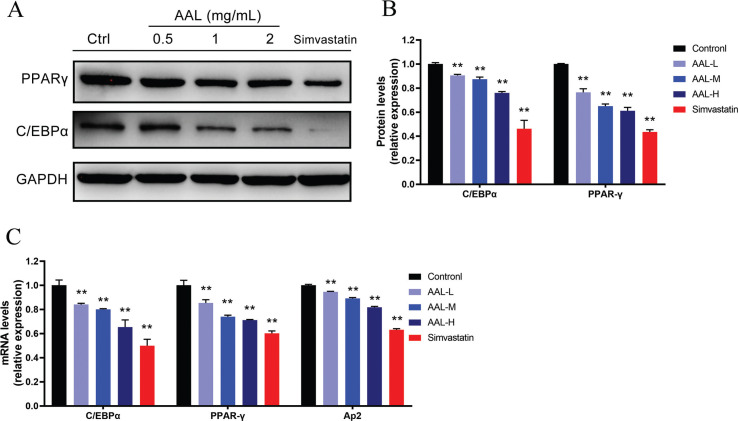
AAL treatment effect on C / EBPα and PPARγ expression in adipogenesis. Representative immunoblots of C/EBPα and PPARγ proteins are shown (A). Relative expression levels of C/EBPα and PPARγ normalized to internal control GAPDH (B). Relative mRNA expression levels of C/EBPα, PPARγ and aP2 normalized to internal control HPRT (C). **P < 0.01 compared with control group. Data are presented as means ± SEM. The concentrations of AAL are 0.5,1,2 mg/mL, respectively, while simvastatin is 10 μmol/L.

PPARγ, C/EBPα, and aP2 play important roles in adipocyte differentiation. In order to investigate the underlying mechanism for the anti-obesity effect of AAL, the expression levels of PPARγ and C/EBPα in differentiated 3T3-L1 cells were studied by using qPCR and Western blot. In Fig. 8, qPCR data showed that compared with the control group, C/EBPα mRNA expression in the AAL-L, AAL-M, AAL-H, and simvastatin groups were reduced by 16, 20, 35, and 50%, respectively (*P* < 0.05). The mRNA expression of PPARγ decreased by 5, 26, 29, and 40%, respectively (*P* < 0.05), and aP2 decreased by 5, 11, 18, and 37%, respectively (*P* < 0.05). Western blot data showed that compared to the control group, the expressions of PPARγ and C/EBPα in the simvastatin group decreased by 60 and 50%, respectively. AAL treatment resulted in decreasing PPARγ and C/EBPα expression, and AAL-L, AAL-M, and AAL-H treatment reduced PPARγ expression levels by 25, 35, and 40% and reduced C/EBPα expression levels by 10, 14 and 24%.

## Discussion

This study was carried out to investigate the potential anti-obese properties of AAL water extract in HFD-induced obese mouse model. Additionally, the potential mechanism of action was investigated in vitro in 3T3-L1 cell line. To our knowledge, this is the first time that AAL has been proved to significantly reduce weight gain and fat pads in obese mice, while hardly affecting animal appetite (Table 1, Fig. 2, and Supplementary Fig. 1). Moreover, AAL significantly reduced serum levels of TG, TC, and LDL-C, as well as ameliorated the accumulation of hepatic lipids, suggesting pharmacological potential to anti-obesity and improving hyperlipidemia. We demonstrate that AAL showed the beneficial effect by downregulating the gene and protein expression of PPARγ and C/EBPα to inhibit the adipocytes differentiation, and reversing intestinal dysbacteriosis induced by HFD.

ALT and AST are specific toxicological indices for liver function text, which usually reveal liver injury (24). The data demonstrated that serum levels of AST and ALT were significantly increased after HFD administration, which were reversed by AAL treatment (Supplementary Fig. 2), suggesting that AAL could relieve the toxicity to the liver function induced by HFD and had no hepatotoxicity.

The epididymal adipose tissue represents subcutaneous fat pad, while the perirenal tissue represents abdominal visceral fat pad. It has been reported that hypotrophy of visceral fat is the risk for cardiovascular diseases development, while subcutaneous fat accumulation is a relatively protective factor which encounters excess energy intake (25). Epididymal and perirenal adipose tissue samples were usually taken to determine the wet weight of the fat pad and body fat ratio in the absence of dual-energy X-ray absorptiometry (DEXA) (26). Compared with the control group, wet weight of the fat pad was much higher than that in the model group, suggesting a successful HFD-induced obesity mouse model. Similar with simvastatin, AAL could significantly reduce body fat and the ratio of body fat in obese mice. In addition, AAL significantly inhibited the expansion of subcutaneous fat and the effects are dose-dependent. Moderate and high-dose group of AAL significantly reduces perirenal fat, while low-dose group of AAL only showed a trend toward decrease. These data demonstrate that AAL is better at inhibiting subcutaneous fat accumulation. These might be the fact that subcutaneous fat loss is much easier than visceral ones due to their differences in natural properties. Nowadays, scientists tended to believe that subcutaneous fat is the intermediate pool between excessed lipid in circulation and visceral fat. Reducing subcutaneous fat pad can effectively inhibit visceral fat expansion, thereby reducing cardiovascular risk (27, 28). Lee’s Index is an effective indicator of obesity. As shown in Table 2, we can also observe significant improvement on Lee’s Index in all dosage groups of AAL, which again verified the anti-obesity effect of AAL.

There are no significant treatment effects of AAL on Liver Index or Kidney Hypertrophy Index. However, serum lipid profiles including TC, TG, and LDL-C are significantly decreased after the AAL treatment in obese mice, while HDL-C increased significantly (Fig. 3). Among all the bioactives being reported in AAL (10, 29–32), botanical extract of flavonoids is supposed to play an important role in blood lipid adjustment (33–35). An inverse relationship between flavonoid consumption and risk factors for heart diseases including improved weight management and improved dyslipidemia, has been established (36, 37). We determined the percentage of total flavonoids by using UV spectrophotometry with rutin as a control component, according to the reported literature (38). Data showed that the total flavonoids in the water extract of AAL for the current sample is 2.4%, which might contribute to lower-hyperlipidemia effect of AAL (data now shown). It is reported that the content of total flavones in AAL varied during different harvest time. Considering the content of active ingredients and biological yield, July is the best time to pick of AAL (29). Further identification is needed to determine which kind of flavonoids is present.

Liver and adipose tissues play important roles in lipid metabolism (39). We tested whether AAL improves fat storage in liver and adipose tissue by analyzing the pathological slices using H–E and ORO staining methods. The liver tissues in the model group developed a high degree of fat accumulation, compared to the control one. The hepatocytes were with severe fat vacuoles, and the infiltration of inflammatory cells and the symptoms of fatty liver were mitigated by varying degrees in simvastatin, AAL-L, AAL-M, and AAL-H groups (Fig. 4A). For the subcutaneous (epididymal) adipose tissue (Fig. 4B), the number of adipocytes in the model group was decreased compared to those of other groups, while the size of individual cells was much larger with a significantly increased diameter, as shown in Fig. 4C and D. Treatment with simvastatin and AAL, especially AAL-M and AAL-H, significantly inhibited the expansion in adipocyte number or size, suggesting that mechanism through which AAL inhibits lipid accumulation might be adipogenesis.

In obesity, adipose tissue accumulation is partly caused by hyperplasia which occurs through adipogenesis, in which preadipocytes differentiate into mature adipocytes. As a nuclear hormone receptor, peroxisome proliferator-activated receptor gamma (PPARγ) regulates cell proliferation and differentiation at the transcriptional level by affecting the function of fatty acids and their derivatives. In this way, activated PPARγ promotes adipocyte differentiation and increases the number of adipocytes and lipid storage in adipose tissue (40, 41). Adipocyte protein 2 (aP2), also called FABP4, regulates adipogenesis by downregulating PPARγ (42). CCAAT/enhancer binding protein alpha (C/EBPα), a member of a sub-family of the basic leucine zipper protein family, activates the differentiation of adipocytes and the transcriptional expression of related genes, which promote the formation of lipid droplets (43, 44). It has been reported that C/EBPα knockout mice have no white adipose tissue in many fat depots in the presence of high serum lipid levels, demonstrating that C/EBPα is essential for terminal differentiation of adipocytes in white adipose tissue (45). Through the intervention and regulation of various stages of lipid droplet formation, the number and volume of adipocytes can be effectively controlled. In our study, AAL-M and AAL-H have similar effect as simvastatin, which down-regulated the gene and protein expression of PPARγ and C/EBPα, verifying that the extract of AAL could inhibit lipid accumulation by modulating adipogenesis (Fig. 8).

**Fig. 9:**
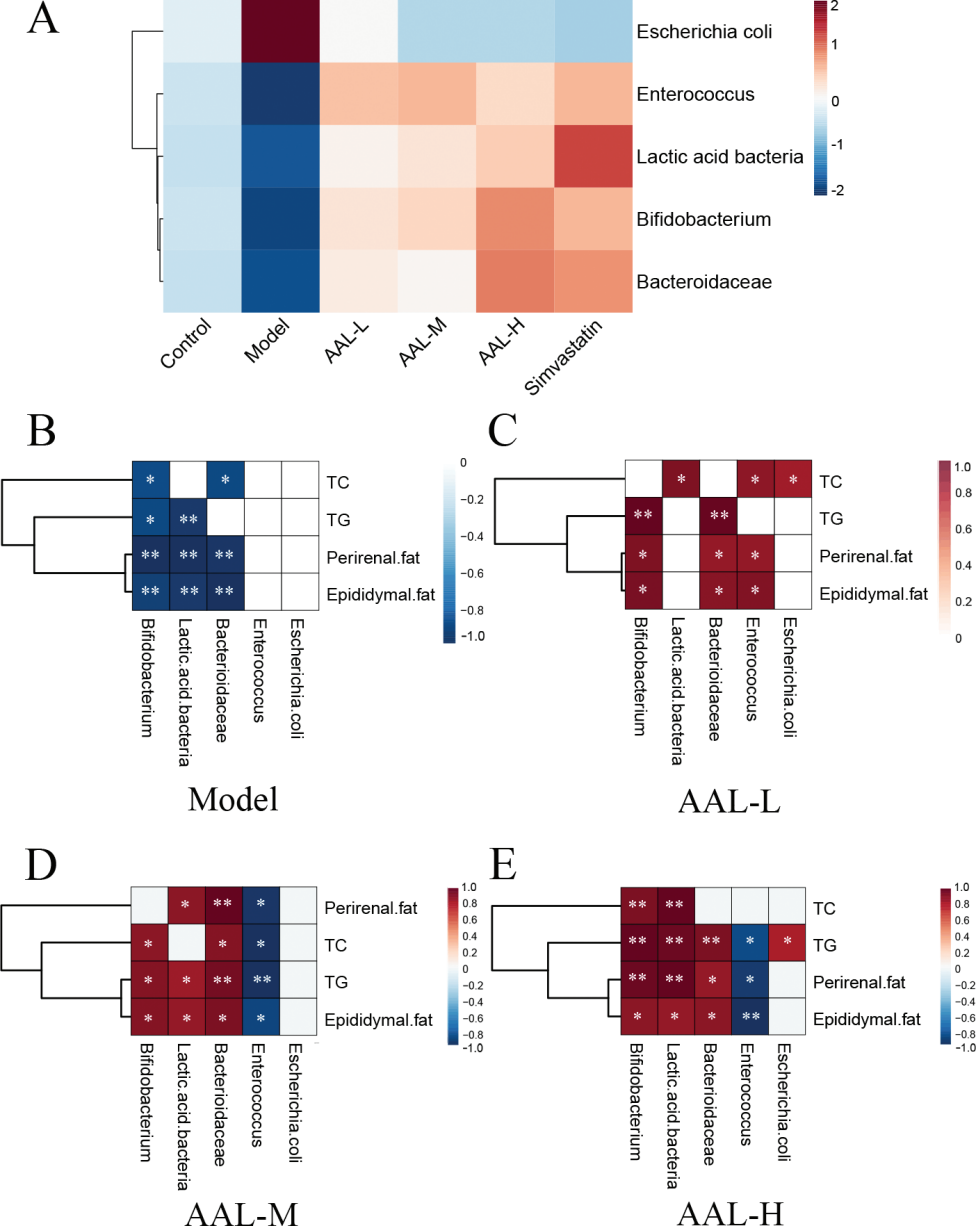
Heat-map between intestinal flora and treatment groups/physiological indexes (fat weight, TC level, and TG level). Heat-map between intestinal flora and treatment groups (A). Correlation analysis between intestinal flora and physiological indexes in model (B), AAL-L (C), AAL-M (D) and AAL-H (E) group. A color key with the correlation coefficient is shown to the right of the heat map; red represents a positive correlation; blue represents a negative correlation. *P < 0.05; **P < 001.

We also studied the effect of AAL on intestinal flora. Compared with the control group, bifidobacterium, lactobacillus, bacteroides, and enterococci were significantly decreased, while *Escherichia coli* increased in the model group (Fig. 8A). Data are consistent with the reports that HFDs induced the gut microbiota dysbiosis and accelerated fat storage (46). AAL increased the number of lactic-acid bacteria, bacteroidaceae, and bifidobacterium in a dose-dependent manner, demonstrating that intestinal flora are the important target of action of AAL. What’s more, bifidobacterium, lactiobcillus, and bacteroidaceae showed strong negative correlation with TC, TG, perirenal, or epididymal fat in the model group, while in AAL treatment group, it tended to be reversed to a significantly positive correlation (Fig. 9B, C, D, and E). It has been reported that HFD contributed to the reduction of relative abundances of bacteroidaceae (47), bifidobacteria, and lactobacillus colonies (48), which could weaken the process of cholesterol conversion and utilization, leading to hyperlipidemia (49–51). Lactobacillus was proved to inhibit HFD-induced obesity by down-regulating PPARγ and C/EBPα (52). These data prompted that intestinal flora might also contribute to the effect of AAL on inhibiting lipid accumulation via suppressing transcriptional regulation factor during adipogenesis. However, we also noticed some interesting data that need further verification that the enterococcus was positively correlated with perirenal fat or epididymal fat in AAL-L group, while in the AAL-M and AAL-H group, it showed the opposite trends (Fig. 9C, D, and E).

## Conclusions

In summary, AAL showed anti-obesity effects by inhibiting lipid accumulation through modulating adipogenesis. In addition, AAL reversed gut microbiota dysbiosis during HFD administration. AAL has the potential to be an effective natural material for anti-obesity. However, more in-depth researches need to be performed in the near future to explore the underlying mechanism.

## Supplementary Material

Extract of *Acalypha australis L.* inhibits lipid accumulation and ameliorates HFD-induced obesity in mice through regulating adipose differentiation by decreasing PPARγ and CEBP/α expression
