# Supplementary material for: Extract of Acalypha australis L. inhibits lipid accumulation and ameliorates HFD-induced obesity in mice through regulating adipose differentiation by decreasing PPARγ and CEBP/α expression
Source: Food Nutr Res. 2021 Mar 1;65:10.29219/fnr.v65.4246. doi: 10.29219/fnr.v65.424 (PMC7955518; doi:10.29219/fnr.v65.424)
Supplement: Extract of Acalypha australis L. inhibits lipid accumulation and ameliorates HFD-induced obesity in mice through regulating adipose differentiation by decreasing PPARγ and CEBP/α expression [file FNR-65-4246-s001.docx]

**Supplementary Table 1**

**Supplementary Table 1. Composition of Food Pellets**

| composition | content (%) | |
| --- | --- | --- |
|  | chow diet | high-fat diet |
| Crude protein（CP） | 18 | 14.184 |
| Crude fat（CF） | 4 | 3.152 |
| Crude fiber（CF） | 5 | 3.94 |
| Crude ash（CA） | 8 | 6.304 |
| water | 10 | 7.88 |
| Lysine（Lys） | 0.82 | 0.64616 |
| Cystine | 0.53 | 0.41764 |
| Constant element | 3.9 | 3.0732 |
| Trace element | 0.02157 | 0.016997 |
| Vitamin | 0.0027 | 0.002128 |
| lard | 0 | 10 |
| Egg yolk powder | 0 | 10 |
| cholesterol | 0 | 1 |
| Bile salt | 0 | 0.2 |

**Supplementary Table 2**

**Supplementary Table 2. Primer pairs used for the real-time quantitative reverse-transcription (RT-qPCR) analysis**

| Gene name | Primer sequence (5′ to 3′) | |
| --- | --- | --- |
| C/EBPα | Forward | TCAGACCAGAAAGCTGAGTTGTG |
|  | Reverse | TGGTCCCCGTGTCCTCCT |
| PPARγ | Forward | TCGCTGATGCACTGCCTATG |
|  | Reverse | GAGAGGTCCACAGAGCTGATT |
| aP2 | Forward | GCTGGTGGTGGAATGTGTTA |
|  | Reverse | AATTTCCATCCAGGCCTCTT |
| HPRT | Forward | TGCTGACCTGCTGGATTACA |
|  | Reverse | TTTATGTCCCCCGTTGACTGA |

**Supplementary Figure 1**


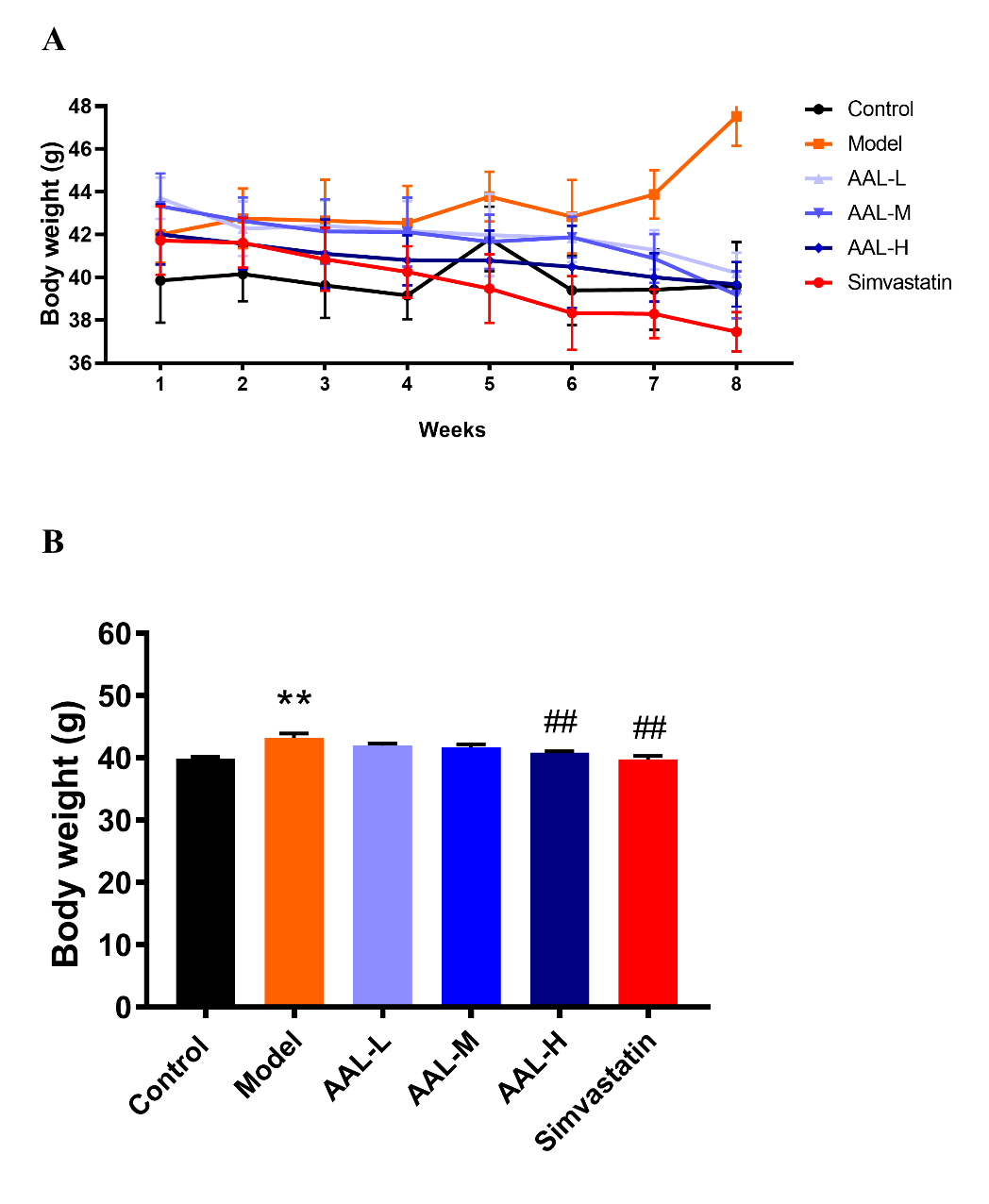


Supplementary Figure 1. Body weight changes (A) during AAL treatment. B showed the body weight between groups after 8-weeks of AAL treatment. *∗∗P* < 0.01 compared with Control group. *##P* < 0.01 compared with Model group. Data are presented as means ± SEM, n = 6.

**Supplementary Figure 2**


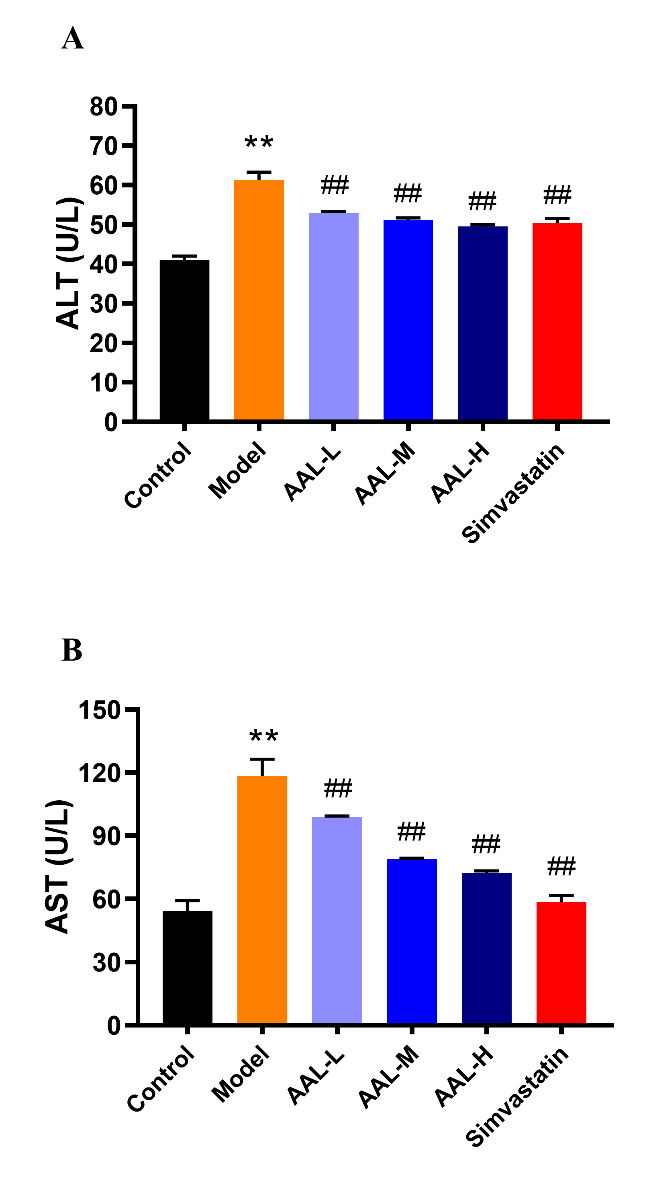


Supplementary Figure 2. *Acalypha australis L.* reduced the increased serum levels of ALT and AST induced by HFD. A for determination of serum levels of alanine aminotransferase (ALT), B for determination of serum levels of aspartate aminotransferase (AST). *∗∗P* < 0.01 compared with Control group. *##P* < 0.01 compared with Model group. Data are presented as means ± SEM, n = 6.
